# SLC15A4 Serves as a Novel Prognostic Biomarker and Target for Lung Adenocarcinoma

**DOI:** 10.3389/fgene.2021.666607

**Published:** 2021-06-08

**Authors:** Hui Huang, Junwei Wang, Shibin Chen, HongJiang He, Yu Shang, Xiaorong Guo, Ge Lou, Jingjing Ji, Mian Guo, Hong Chen, Shan Yu

**Affiliations:** ^1^Department of Operating Room, The Second Affiliated Hospital of Harbin Medical University, Harbin, China; ^2^Department of Pulmonary and Critical Care Medicine, The Second Affiliated Hospital of Harbin Medical University, Harbin, China; ^3^Medical Research Center, Beijing Chao-Yang Hospital, Capital Medical University, Beijing, China; ^4^Department of Head and Neck Surgery, Harbin Medical University Cancer Hospital, Harbin, China; ^5^Department of Pulmonary and Critical Care Medicine, The First Hospital of Harbin, Harbin, China; ^6^Department of Pathology, The Second Affiliated Hospital of Harbin Medical University, Harbin, China; ^7^Department of Neurosurgery, The Second Affiliated Hospital of Harbin Medical University, Harbin, China

**Keywords:** SLC15A family, lung cancer, prognostic biomarker, NSCLC, peptide transporter

## Abstract

**Background:**

SLC15A family members are known as electrogenic transporters that take up peptides into cells through the proton-motive force. Accumulating evidence indicates that aberrant expression of SLC15A family members may play crucial roles in tumorigenesis and tumor progression in various cancers, as they participate in tumor metabolism. However, the exact prognostic role of each member of the SLC15A family in human lung cancer has not yet been elucidated.

**Materials and Methods:**

We investigated the SLC15A family members in lung cancer through accumulated data from TCGA and other available online databases by integrated bioinformatics analysis to reveal the prognostic value, potential clinical application and underlying molecular mechanisms of SLC15A family members in lung cancer.

**Results:**

Although all family members exhibited an association with the clinical outcomes of patients with NSCLC, we found that none of them could be used for squamous cell carcinoma of the lung and that SLC15A2 and SLC15A4 could serve as biomarkers for lung adenocarcinoma. In addition, we further investigated SLC15A4-related genes and regulatory networks, revealing its core molecular pathways in lung adenocarcinoma. Moreover, the IHC staining pattern of SLC15A4 in lung adenocarcinoma may help clinicians predict clinical outcomes.

**Conclusion:**

SLC15A4 could be used as a survival prediction biomarker for lung adenocarcinoma due to its potential role in cell division regulation. However, more studies including large patient cohorts are required to validate the clinical utility of SLC15A4 in lung adenocarcinoma.

## Introduction

The solute carrier (SLC) group of membrane transport proteins contains over 400 members classified into 65 families (Hediger et al., 2004). Most solute carrier proteins are inserted in the cell membrane and are responsible for the transportation of intracellular and extracellular metabolites (Perland and Fredriksson, 2017).

Solute carrier family 15 members are proteins encoded by SLC15A genes, and their oligopeptide transporter activity is pH-dependent. With this feature, the members of the SLC15A family are also known as pH-sensing regulatory factors of peptide transporters (PEPTs) (Capo et al., 2017). In mammalian cells, four gene members, SLC15A1 (PEPT1), SLC15A2 (PEPT2), SLC15A3 (PHT2), and SLC15A4 (PHT1), comprise the SLC15A family. SLC15A1 and SLC15A2 were first discovered in the small intestine and kidney, and their physiological functions have been well investigated (Huo et al., 2017). In addition to taking up peptides, SLC15A3 and SLC15A4 were able to transport histidine across cell membranes with the alias “peptide-histidine transporter” (Song et al., 2018). However, these proteins only share less than 20% amino acid homology with SLC15A1 and SLC15A2 (Sakata et al., 2001).

In recent years, the importance of cellular metabolism in cancer has aroused interest for anticancer therapy. Since the discovery of aberrant peptide transporter expression in cancers, these transporters have been considered an important switch for regulating cancer metabolism and anticancer bioactive peptide delivery. Moreover, it is promising to utilize peptide transporters for drug delivery in antitumor therapy. Although intensive studies have focused on these transporters since they were discovered, their exact pathological functions have not yet been revealed in cancers.

Lung cancer is the leading cause of cancer death among both men and women worldwide (Torre et al., 2016). Lung cancer currently includes small-cell lung carcinoma and non-small-cell lung carcinoma (NSCLC). Non-small-cell lung carcinomas are further classified into large cell carcinoma (LCC), adenocarcinoma (AD), and squamous cell carcinoma (SCC) (Chi et al., 2017). To date, predictive biomarkers for lung cancer clinical outcomes have been reported. With the development of genomic network analysis from large databases, we may better understand the underlying biological functions of biomarkers (Yu et al., 2019). As our knowledge of the SLC15A family in human malignancies is largely unexplored, in our present study, we performed a comprehensive analysis to investigate the potential of SLC15A family members as biomarkers for lung cancer and the underlying molecular mechanisms.

## Materials and Methods

### Pan-Cancer and Lung Cancer Analysis of the SLC15A Family by Oncomine

Oncomine^1^ is a gene expression microarray combination platform (Rhodes et al., 2004). The online access version was used to determine the transcriptome expression profile of each SLC15A family member in different kinds of malignancies and paired normal tissues. “SLC15A” and “cancer vs. normal tissue analysis” were used as search keywords. The cancer types with t-test *P*-values and expression fold changes were obtained. *P* < 0.05, twofold change and gene rank within the top 10% were input for study selection for both DNA and mRNA studies. Study numbers meeting our threshold are displayed in a heatmap. The TCGA lung cancer dataset in Oncomine was also extracted to obtain the detailed expression of SLC15A family members in lung cancer subtypes. A boxplot was applied by log2 median-centered ratio.

### Expression Profile of SLC15A Family Members in Lung Cancer and Normal Tissue by GEPIA

We extracted SLC15A family RNA-seq data in both lung cancer and normal tissue from TCGA and GTEx by Gene Expression Profiling Interactive Analysis (GEPIA) (Tang et al., 2017). A heatmap was plotted to demonstrate the RNA-seq data among SLC15A family members using log2 (TPM + 1) in both LUAD and LUSC. Additionally, a box plot of individual patients in both LUAD and LUSC was used to show the expression difference between tumor and normal tissue. In addition, the expression of SLC15A family members in the NSCLC major stage was compared and displayed by violin plots with one-way ANOVA.

### SLC15 Family Member Mutations in the TCGA Dataset

cBioportal is based on the Memorial Sloan Kettering Cancer Center and provides comprehensive analyses of cancer genomics and clinical profiles of different cancer types in The Cancer Genome Atlas (TCGA)^2^ (Cerami et al., 2012; Gao et al., 2013). The online database is accessed for cancer genomics mutation analysis by querying formal gene names in lung cancer datasets. The dataset used was Non-Small-Cell Lung Cancer (MSKCC, J. Clin. Oncol. 2018) (Rizvi et al., 2018).

### Overall Survival Kaplan−Meier Analysis for NSCLC and Subtypes

To investigate the independent prognostic value of SLC15A family members in lung cancer, Kaplan-Meier plotter was used^3^ (Nagy et al., 2018). The Kaplan-Meier plotter including 3,452 lung cancer patient survival data points is capable of assessing the effect of 54k genes on survival in 21 cancer types. The NSCLC and lung adenocarcinoma (LUAD) and lung squamous cell carcinoma (LUSC) subtype patients in overall survival analysis were assessed by median signal expression. A log-rank *P*-value < 0.05 was considered statistically significant. As SLC15A2 (PEPT2) has been widely studied in cancer fields, we only chose SLC15A4 for molecular mechanism downstream analysis.

### Immunohistochemistry (IHC) From HPA and Clinical Patients

The Human Protein Atlas (HPA) is an online web-access antibody-based proteomics database of human disease and normal tissues^4^ (Thul et al., 2017). In our study, representative SLC15A4 IHC staining with the antibody HPA016713 was obtained from HPA in normal lung tissue and lung adenocarcinoma. In addition, IHC staining was performed in 20 cases of AD for validation in clinical practice. Informed consent was signed by all patients, and the study was approved by the Ethics Committee of Harbin Medical University. All tissue samples were obtained from leftover tumors from the pathology department after diagnosis and processed anonymously in accordance with ethics and law. The slides were soaked twice in 100% xylene for 5 min and dehydrated in different concentrations of ethanol (100%, 5 min; 100%, 5 min; 90%, 5 min; 80%, 5 min; and 70%, 5 min). The slides were washed three times with pure water for 3 min and placed into boiling citric acid repair solution for 15 min. After cooling to room temperature, endogenous peroxidase blocking solution was applied to each slide for 10 min and then washed three times with PBS for 5 min. Primary antibody (1:100; Biosource) was used and placed at 4°C overnight. The following day, the slides were washed with PBS for 5 min and incubated with the secondary antibody (Maxin, China) at room temperature for 60 min followed by three washes with PBS for 5 min. Diluted DAB (Maxin, China) solution was added for 3 min and stopped with tap water for 15 min followed by the addition of hematoxylin for 2 min. Finally, the slides were immersed in 0.25% hydrochloric acid alcohol for 2 s and washed with water for 2 min.

### Coexpression of the SLC15A4 Gene in the TCGA Lung Adenocarcinoma RNA-Seq Dataset and Functional Enrichment Analysis

LinkedOmics^5^ is a unique online web-accessible gene analysis tool for understanding large-scale cancer omics data. It contains multiomics data and clinical information for 32 major cancer types (Vasaikar et al., 2018). We used lung adenocarcinoma (LUAD) (TCGA-LUAD) from UNC University of North Carolina RNA-seq data through the HiSeq RNA platform running the Firehose_RSEM_log2 pipeline. A Pearson correlation test was performed. A volcano plot and heatmap with the top 50 positively and negatively regulated genes were used to display SLC15A4 Pearson correlation coefficient gene expression.

All positive genes with a Pearson correlation score > 0.3 and negative genes with a Pearson correlation score < −0.2 were selected for Gene Ontology (GO) terms (biological process), Reactome pathways and Kyoto Encyclopedia of Genes and Genomes (KEGG) pathway analysis by Metascape^6^ (Zhou et al., 2019), which is a tool for gene annotation with a list of genes of interest. Moreover, we applied gene set enrichment analysis (GSEA) for all correlated genes with GO biological processes. Parameters for the enrichment analysis were set as a minimum number of hit genes in category 3 and 500 permutations. The top 25 enriched GO terms listed by the normalized enrichment score (NES) with FDR are plotted in a bar chart. The highest positive and negative enriched results are displayed.

## Results

### SLC15A Family Member Transcript Expression Studies in Pan-Cancer

**FIGURE 1:**
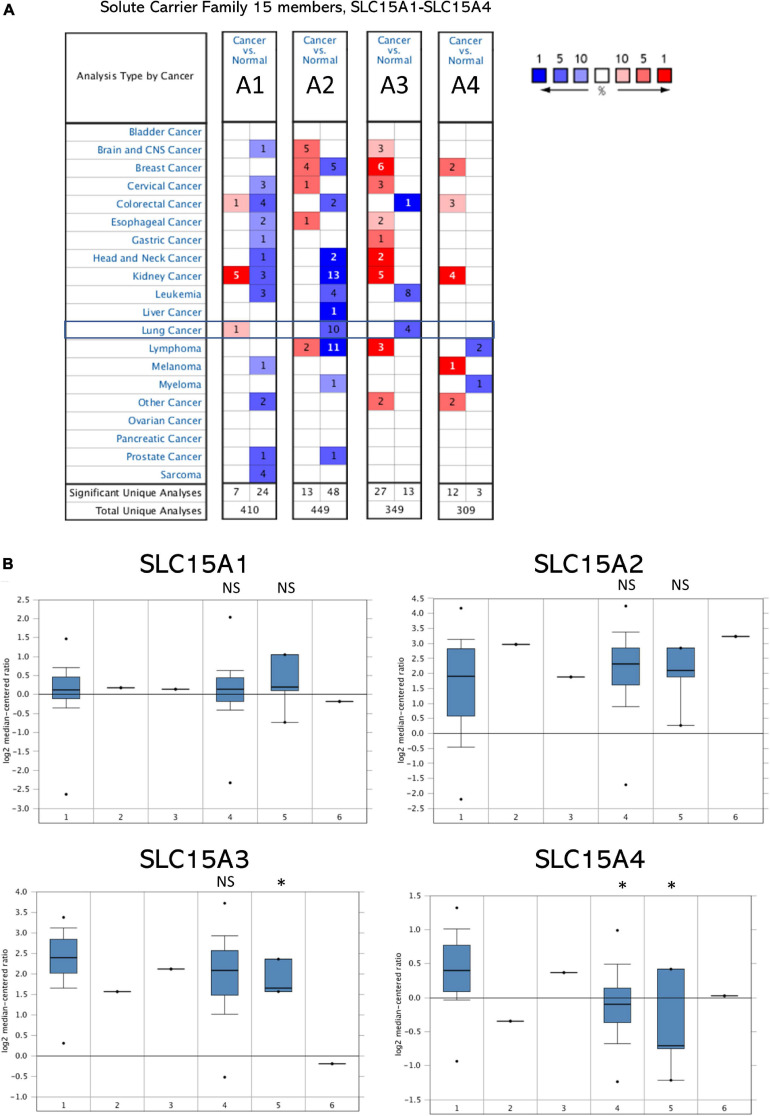
**(A)** Pan-cancer analysis of SLC15A family genes within the Oncomine database. Lung cancer is indicated in the frame. Cells on red background with numbers show research within our selection standard in cancer tissue; blue in normal tissues. A1 = SLC15A1, A2 = SLC15A2, A3 = SLC15A3, and A4 = SLC15A4. Blue color indicates down-regulated genes percent and red color is for up-regulated genes percent. Number in the cell is for the research cases matched with our selection. **(B)** The RNA-Seq profile of SLC15A family members from TCGA lung cancer with detailed subtypes. 1, lung adenocarcinoma (30 cases); 2, lung adenocarcinoma, mixed subtypes (1 case); 3, Lung clear cell adenocarcinoma (1 case); 4, squamous cell lung carcinoma (*n* = 149); 5, squamous cell carcinoma, basaloid variant (*n* = 5); 6, squamous cell lung carcinoma, papillary variant (*n* = 1). NS, not statistically significant. ^∗^*P* value < 0.05.

SLC15A family members from SLC15A1 to SLC15A4 were searched in 20 major cancers through the Oncomine database. The Oncomine database contains a total of 410, 449, 349, and 309 distinct studies with SLC15A family genes (Figure 1A). According to our selection standard for those cancers with unique analysis, the studies included were SLC15A1 (7:24), SLC15A2 (13:48), SLC15A3 (27:13), and SLC15A4 (12:3). Regarding lung cancer, only one study showed increased SLC15A1 expression compared with that in normal tissue. SLC15A2 and SLC15A3 both showed downregulated patterns in 10 and 4 studies, respectively. However, for SLC15A4, there was no significant research meeting our selection criteria in the Oncomine database for lung cancer. For other major cancer types, SLC15A1 was significantly increased in kidney cancer in 5 cases, with 3 opposite results. However, in most cancer types, SLC15A1 showed relatively lower expression. SLC15A2 also displayed a pan-cancer decreased pattern, especially in kidney, lung and lymphoma. SLC15A3 and SLC15A4 were different from SLC15A1 and SLC15A2, with increased patterns in pan-cancer compared with normal tissue. SLC15A2 had a controversial expression pattern with SLC15A3 across the pan-cancer study, where SLC15A1 showed a down-regulated expression in most of solid cancers and SLC15A4 displayed an up-regulated pattern.

### SLC15A Family Association With Lung Cancer Subtypes, Stages and Mutations

**FIGURE 2:**
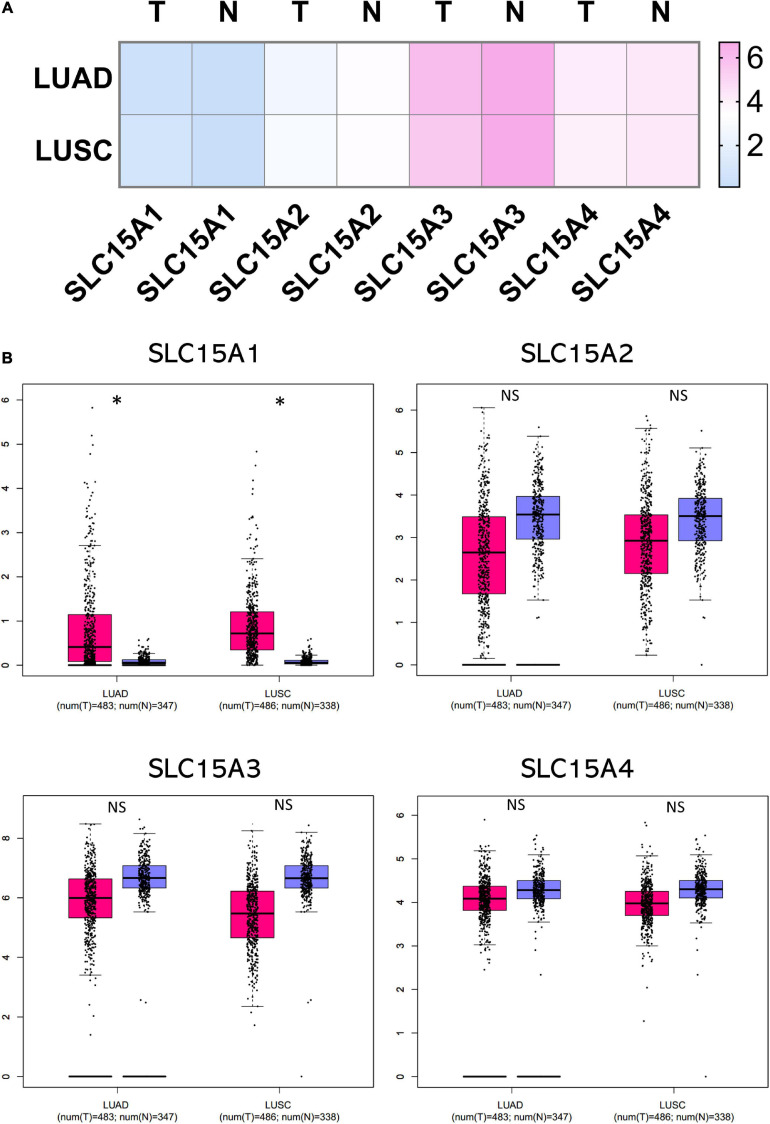
Comparison of normalized SLC15A family member expression levels in tumor samples vs. normal tissue. The cancer abbreviation name is shown according to TCGA study abbreviation (LUAD = lung adenocarcinoma; LUSC = lung squamous cell carcinoma). **(A)** Heatmap of SLC15A family relative expression after normalization by log2(TPM + 1) for log-scale. T, tumor; N, normal tissue. Light blue indicates lower expression and pink color is for relative higher expression across the SLC15A family. **(B)** Box plot of RNA-seq expression details for individual patients and average. The Y axis is using TPM. Pink is for the tumor samples, and purple is for the normal lung tissue. T, tumor (Pink); N, normal tissue (Purple); NS, not statistically significant. ^∗^*P* value < 0.05.

The microarray expression from the TCGA lung cancer dataset obtained from Oncomine showed the relative mRNA expression within the SLC15A family in different lung cancer subtypes (Figure 1B). We found that there was little difference between lung adenocarcinoma and squamous cell carcinoma in SLC15A1, SLC15A2 and SLC15A3. For SLC15A4, the expression in lung adenocarcinoma (30 study cases) was much higher than that in lung squamous cell carcinoma (149 study cases) (Figure 1B). In addition, the heatmap showed that SLC15A3 had the highest RNA-seq reads, followed by SLC15A4 (Figure 2A). SLC15A1 and SLC15A2 were relatively lower expressed within the SLC15A family compared with SLC15A3 and SLC15A4. Importantly, SLC15A1 expression was lower than 1 transcript per kilobase million (TPM). Although SLC15A2 was also decreased vs. normal tissue, the RNA-seq TPM for SLC15A2 was quite low, along with SLC15A1, implying difficulty for clinical usage. For the expression between the lung cancer tissue and normal control tissue (*n* = 347), SLC15A3 and SLC15A4 were both decreased in lung adenocarcinoma (*n* = 483), and squamous cell carcinoma (*n* = 338) (Figure 2B). Moreover, the major stage analysis for lung cancer showed a negative result, as there were no significant average expression differences among the four major lung cancer stages for all SLC15A family members. The F test values are indicated in Supplementary Figure 1A. Mutation analysis showed that SLC15A2 had the highest total mutation rate, up to 5%, in the TCGA NSCLC cohort. SLC15A4 had the highest rate of deep deletion in the cohort, which was consistent with the decreased mRNA expression pattern in lung cancer patients (Supplementary Figure 1B). In summary, SLC15A family gene expressions were not associated with lung cancer stages and the mutation rates were also low. Only SLC15A1 had a different expression between the normal lung tissue and tumor samples, its transcription expression was very low (TPM < 1).

### Independent Prognostic Value of SLC15A Family Members by Kaplan-Meier Analysis

**FIGURE 3:**
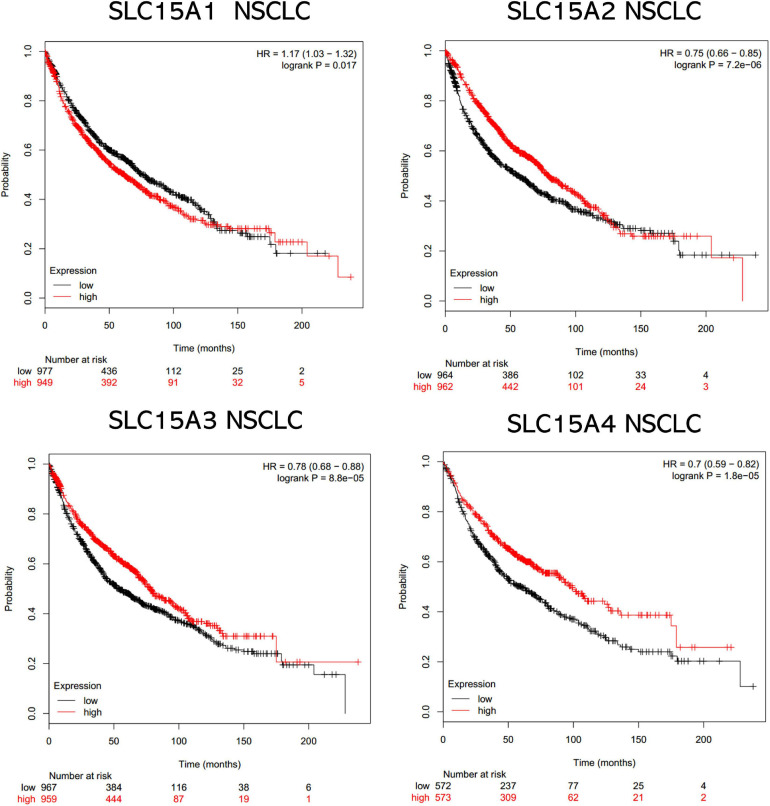
The prognostic values of the SLC15A family members in NSCLC. A log rank *p* value < 0.05 was considered statistically significant. The median expression level was set as the cutoff for the KM plot. The red line indicates high expression, and the blue line indicates low expression. NSCLC, non-small-cell lung carcinoma.

**FIGURE 4:**
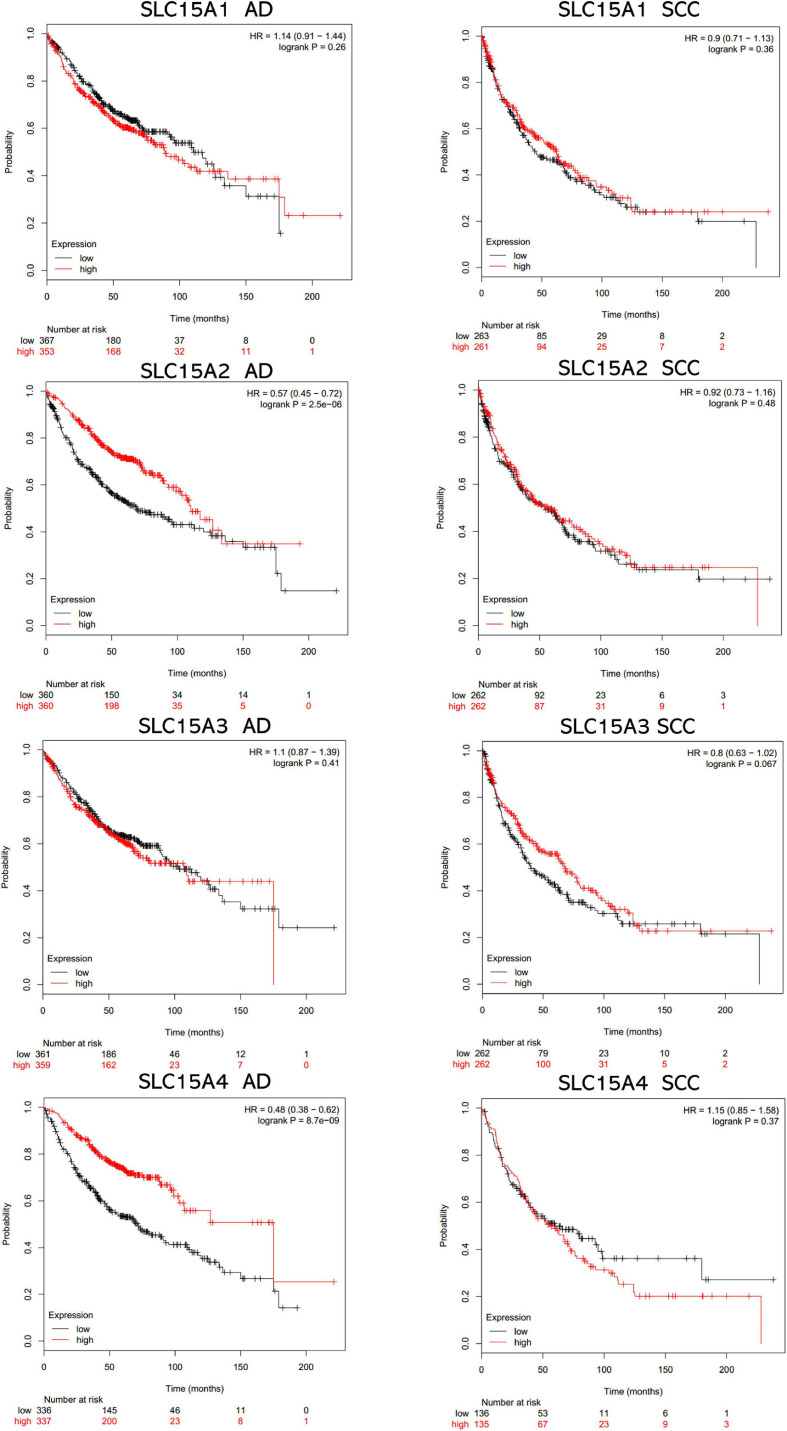
The prognostic values of SLC15A family members in lung cancer major subtypes (AD, adenocarcinoma; SCC, squamous cell carcinoma). The median expression level was set as the cutoff for the KM plot. The red line indicates high expression, and the blue line indicates low expression.

The overall survival data of NSCLC patients from all validation cohorts in the KM plotter database were obtained for Kaplan-Meier analysis of SLC15A family members (Figure 3). A total of 1962 lung cancer cases were divided into high and low expression groups by the median signal of SLC15A1 with the 207254_at probe. The high expression group for SLC15A1 showed a shorter clinical survival time with HR = 1.17 (1.03–1.32) and log rank *P*-value = 0.017. For SLC15A2 (Probe ID: 205316_at), higher expression displayed a better survival rate with HR = 0.75 (0.66–0.85) and log rank *P*-value = 7.2e-06. SLC15A3 (probe ID: 219593_at) also demonstrated the same clinical outcome prediction pattern as SLC15A2, with HR = 0.78 (0.68–0.88) and log rank *P*-value = 8.8e-05. A total of 1145 NSCLC patients with SLC15A4 (probe ID: 225057_at) gene expression were used for overall survival analysis. Higher expression of SLC15A4 also showed a better survival rate with HR = 0.7 (0.59–0.82) and log rank *P*-value = 1.8e-05. We further analyzed the potential biomarker utility in major subtypes (adenocarcinoma and squamous cell carcinoma) of NSCLC for SLC15A family members (Figure 4). Our results clearly showed that only SLC15A2 and SLC15A4 could be used as clinical outcome prediction biomarkers for overall survival rate in lung adenocarcinoma with HR = 0.57 (0.45–0.72); log rank *P*-value = 2.5e-06 and HR = 0.4 (0.38–0.62); log rank *P*-value = 8.7e-09, respectively. However, none of the SLC15A family members could be used to evaluate SCC patient outcomes. SLC15A2 has been widely studied and is expressed at relatively low levels in lung carcinoma. We only selected SLC15A4 for additional GO and KEGG analysis. Briefly, all genes in SLC15A family could be used as clinical outcome prediction biomarkers in NSCLS, while SLC15A2 and SLC15A4 are with favorable prognostic value in lung adenocarcinoma.

### IHC Pattern for SLC15A4

**FIGURE 5:**
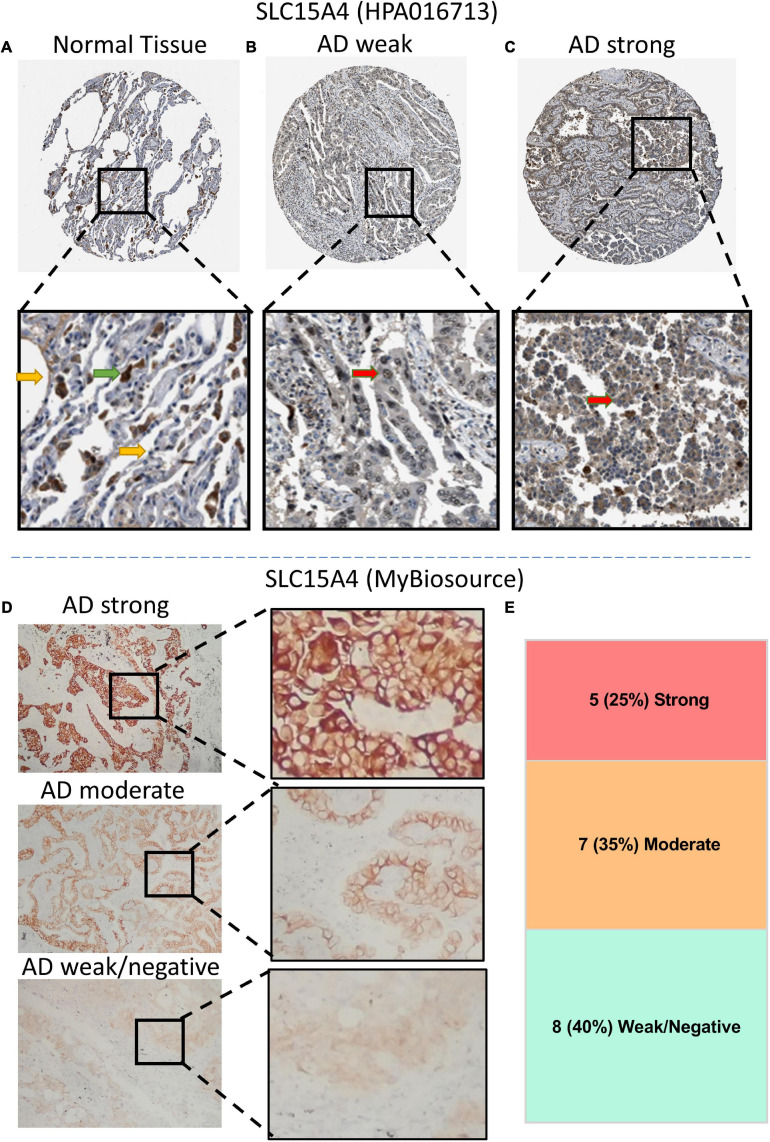
Immunochemistry (IHC) of SLC15A4 in normal lung tissue and lung adenocarcinoma using HPA images (antibody HPA016713) and clinical patients (antibody MyBiosource). **(A)** Normal lung tissue. Positive staining was located mainly in macrophage cells. The green arrow indicates the macrophages, while the yellow arrow points at the normal pneumocytes. **(B,C)** Representative IHC with weak and strong staining in the cytoplasm of lung adenocarcinoma. Black rectangle indicates the zoomed zone in the picture. The red arrow indicates the lung cancer cells. **(D)** Differential expression of IHC intensity in lung adenocarcinoma. SLC15A4 shows a membrane and plasma staining pattern at a 1:100 primary antibody dilution. **(E)** The distribution of IHC intensity in 20 cases of lung adenocarcinoma staining by SLC15A4 antibody.

According to our KM overall survival analysis, SLC15A4 is sensitive and is considered a prognostic biomarker in lung adenocarcinoma. We validated its protein expression pattern from HPA. Normal lung tissue displayed strong staining of macrophages in both the cytoplasm and membrane. Pneumocytes showed low to moderate intensity in the unclear region (Figure 5A). In lung adenocarcinoma, the representative staining of SLC15A4 was weak in the nucleus and cytoplasm (Figure 5B). Strong expression of SLC15A4 always gained positive intensity in the cytoplasm (Figure 5C). For clinical sample validation, we collected 20 AD patients for SLC15A4 IHC staining using a primary antibody purchased from BioSource. The staining pattern was similar to that of HPA, which clearly showed positive staining in both the cytoplasm and membrane (Figure 5D). In our patient cohort, there were five cases (25%) with strong staining, 7 cases (35%) with moderate IHC intensity, and 8 cases (40%) with weak/negative staining (Figure 5E). Our IHC staining result displayed that SLC15A4 antibody could be used as a prognostic prediction marker by its staining intensities.

### Correlation Gene Identification From the TCGA RNA-Seq Dataset

**FIGURE 6:**
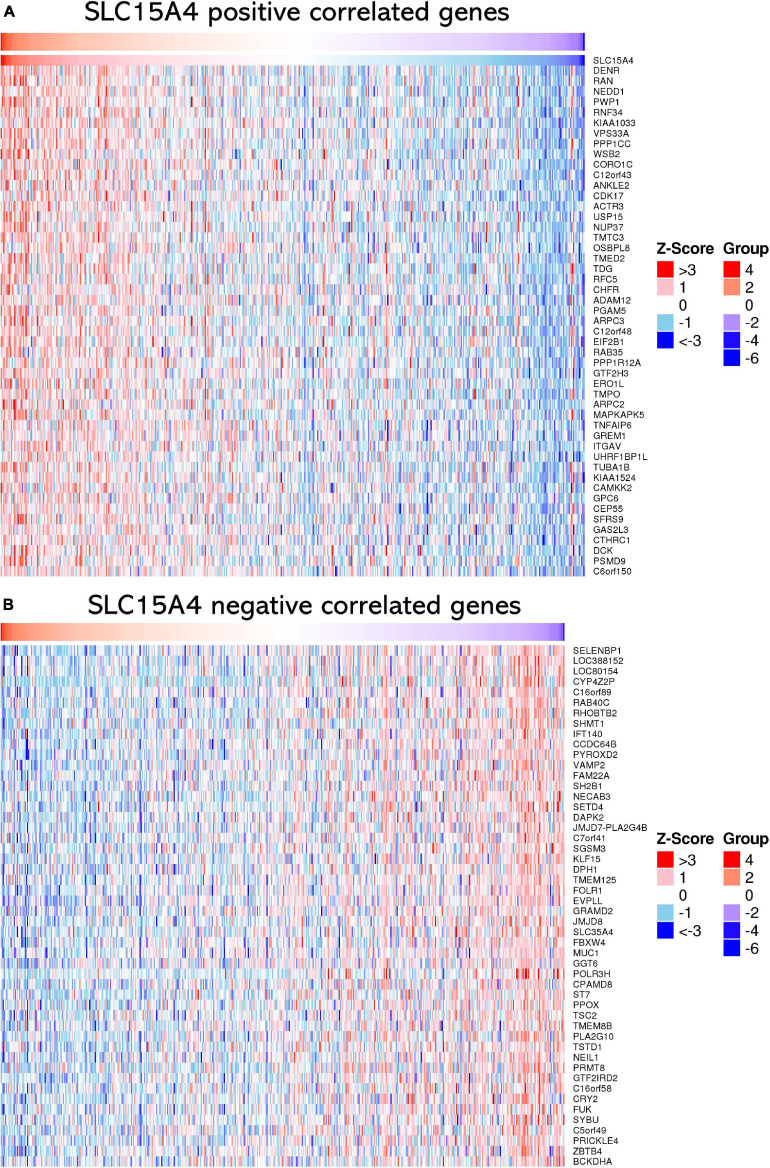
Heatmap of correlated genes of SLC15A4 from TCGA-LUAD using RNA-seq data normalized by Z-score. Blue indicates negatively correlated expressed genes, and red indicates positively correlated expressed genes. **(A)** Heatmap of the top 50 positively correlated genes of SLC15A4 from TCGA-LUAD after normalization. **(B)** Heatmap of the top 50 negatively correlated genes of SLC15A4 from TCGA-LUAD after normalization. Red indicates cases with higher expression, and blue indicates cases with lower expression.

The top 50 positively correlated genes and top 50 negatively correlated genes were plotted in a heatmap (Figures 6A,B). All correlated genes were also plotted into a volcano plot to display the distribution of genes from TCGA lung adenocarcinoma RNA-seq data (Supplementary Figure 2). The top 10 positively correlated genes were DENR (Pearson CC = 0.58); KIAA1033 (Pearson CC = 0.55); ZNF268 (Pearson CC = 0.55); GTF2H3 (Pearson CC = 0.55); CAMKK2 (Pearson CC = 0.55); NEDD1 (Pearson CC = 0.55); ANKLE2 (Pearson CC = 0.54); CHFR (Pearson CC = 0.53); RNF34 (Pearson CC = 0.53); OSBPL8 (Pearson CC = 0.52); and VPS33A (Pearson CC = 0.52). The top 10 negatively correlated genes were ABHD14A (Pearson CC = −0.38); NDUFA2 (Pearson CC = −0.36); LSMD1 (Pearson CC = −0.35); PHPT1 (Pearson CC = −0.34); FOLR1 (Pearson CC = −0.34); SFTA2 (Pearson CC = −0.33); TSTD1 (Pearson CC = −0.33); ATPIF1 (Pearson CC = −0.33); DPM3 (Pearson CC = −0.33); RILP (Pearson CC = −0.33); and NDUFA7 (Pearson CC = −0.32). Those findings suggest that SLC15A4 may major as a gene activator to up-regulate correlated genes than down-regulated gene expression, due to | Pearson correlation count| > 0.3.

3.6 Enrichment analysis with correlated genes for SLC15A4

To understand the biological functions underlying the positively and negatively correlated genes of SLC15A4, GO biological function, Reactome Gene Sets and KEGG pathway enrichment analysis were conducted.

**FIGURE 7:**
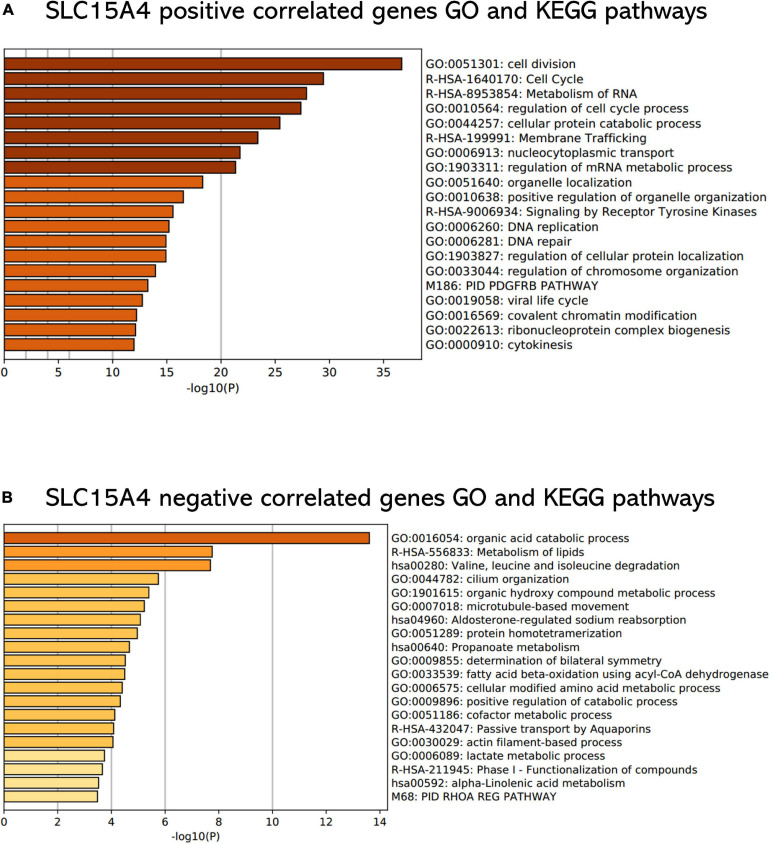
Functional enrichment analysis of Gene Ontology terms and KEGG pathways. **(A)** Enrichment analysis of positively correlated genes with Pearson correlation > 0.3. **(B)** Enrichment analysis of negatively correlated genes with Pearson correlation < –0.2. The *P* value was calculated and sorted with -log10(P). Dark red indicates the smallest *P* value.

**FIGURE 8:**
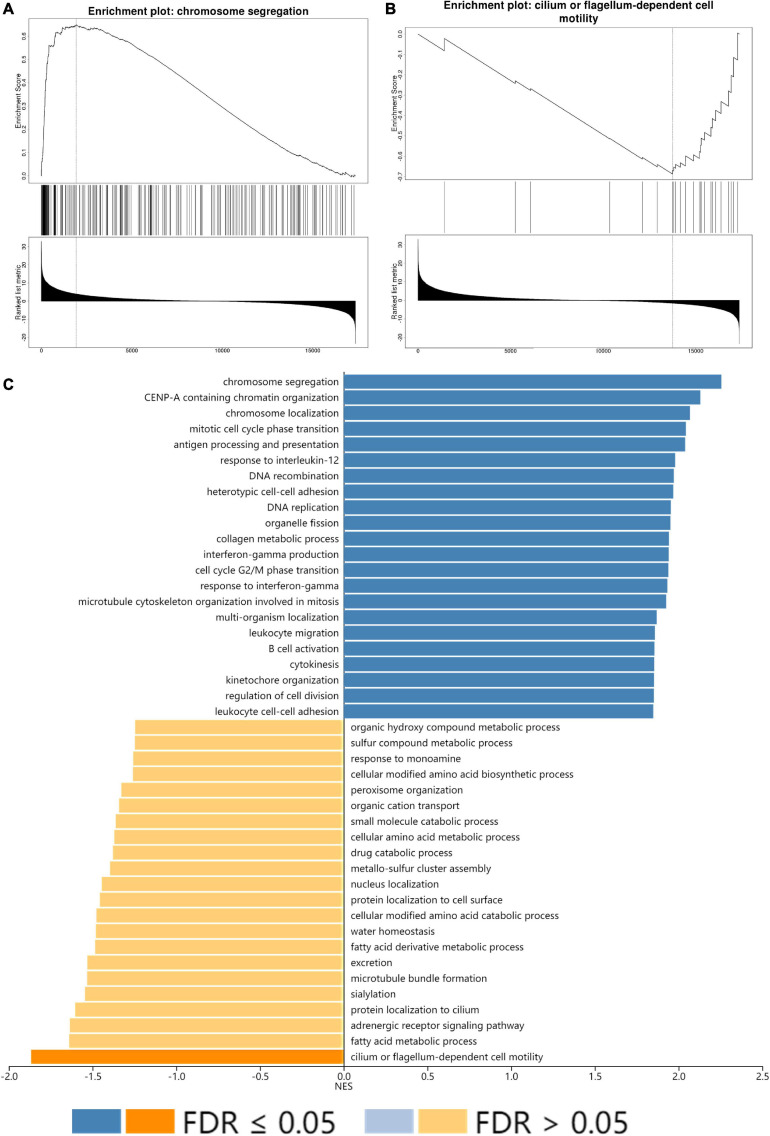
GSEA enrichment analysis of SLC15A4. **(A)** Highest positive Net Enrich Score (NET). **(B)** Lowest negative Net Enrich Score (NET). **(C)** GSEA result of the top 22 positively and negatively enriched GO terms with FDR (false discovery rate). The blue bar indicates positively enriched terms, and the yellow bar represents negatively enriched terms.

All GO terms and KEGG pathways enriched by positively correlated genes are shown in Figure 7A, and negatively correlated genes are shown in Figure 7B. The top 20 GO terms and KEGG pathways for SLC15A4 are listed by -log10(*p*-value). The positively enriched genes were GO:0051301 cell division log10(*p*-value) = −36.67; R-HSA-1640170 Cell Cycle log10(*p*-value) = −29.45; R-HSA-8953854 Metabolism of RNA log10(*p*-value) = −27.89; GO:0010564 regulation of cell cycle process log10(*p*-value) = −27.37; GO:0044257 cellular protein catabolic process log10(*p*-value) = −25.42; R-HSA-199991 Membrane Trafficking log10(*p*-value) = −23.40; GO:0006913 nucleocytoplasmic transport log10(*p*-value) = −21.75; GO:1903311 regulation of mRNA metabolic process log10(*p*-value) = −21.34; GO:0051640 organelle localization log10(*p*-value) = −18.31; GO:0010638 positive regulation of organelle organization log10(*p*-value) = −16.52; R-HSA-9006934 Signaling by Receptor Tyrosine Kinases log10(*p*-value) = −15.57; GO:0006260 DNA replication log10(*p*-value) = −15.20; GO:0006281 DNA repair log10(*p*-value) = −14.92; GO:1903827 regulation of cellular protein localization log10(*p*-value) = −14.91; GO:0033044 regulation of chromosome organization log10(*p*-value) = −13.94; M186 PID PDGFRB PATHWAY log10(*p*-value) = −13.24; GO:0019058 viral life cycle log10(*p*-value) = −12.74; GO:0016569 covalent chromatin modification log10(*p*-value) = −12.20; GO:0022613 ribonucleoprotein complex biogenesis log10(*p*-value) = −12.11 and GO:0000910 cytokinesis log10(*p*-value) = −11.97. The negatively enriched genes were GO:0016054 organic acid catabolic process log10(*p*-value) = −13.61; R-HSA-556833 Metabolism of lipids log10(*p*-value) = −7.75; hsa00280 Valine, leucine and isoleucine degradation log10(*p*-value) = −7.69; GO:0044782 cilium organization log10(*p*-value) = −5.75; GO:1901615 organic hydroxy compound metabolic process log10(*p*-value) = −5.40; GO:0007018 microtubule-based movement log10(*p*-value) = −5.23; hsa04960 Aldosterone-regulated sodium reabsorption log10(*p*-value) = −5.08; GO:0051289 protein homotetramerization log10(*p*-value) = −4.96; hsa00640 Propanoate metabolism log10(*p*-value) = −4.67; GO:0009855 determination of bilateral symmetry log10(*p*-value) = −4.52; GO:0033539 fatty acid beta-oxidation using acyl-CoA dehydrogenase log10(*p*-value) = −4.50; GO:0006575 cellular modified amino acid metabolic process log10(*p*-value) = −4.40; GO:0009896 positive regulation of catabolic process log10(*p*-value) = −4.33; GO:0051186 cofactor metabolic process log10(*p*-value) = −4.13; R-HSA-432047 Passive transport by Aquaporins 5 log10(*p*-value) = −4.08; GO:0030029 actin filament-based process log10(*p*-value) = −4.06; GO:0006089 lactate metabolic process log10(*p*-value) = −3.74; R-HSA-211945 Phase I - Functionalization of compounds log10(*p*-value) = −3.67; hsa00592 alpha-Linolenic acid metabolism log10(*p*-value) = −3.52; and M68 PID RHOA REG PATHWAY log10(*p*-value) = −3.48. Moreover, the GSEA results for the GO biological process of SLC15A4 focused on chromosome segregation and cilium- or flagellum-dependent cell motility (Figures 8A,B). Detailed results of the top enriched GO terms from the GSEA test are summarized with FDR (Figure 8C) and listed in Table 1. Those findings highlight that SLC15A4 plays critical role in cancer cell proliferation through regulating cell cycle related pathway. In addition, the metabolic pathways including fatty acids and collagens are future directions for biological function validation.

**TABLE 1:** GSEA result for GO enrichment.

Gene set	Description	ES	NES	*P* Value	FDR
GO:0007059	Chromosome segregation	0.649203	2.255743	<0.001	<1.00E-05
GO:0061641	CENP-A containing chromatin organization	0.868006	2.130581	<0.001	<1.00E-05
GO:0050000	Chromosome localization	0.695858	2.06804	<0.001	<1.00E-05
GO:0044772	Mitotic cell cycle phase transition	0.570596	2.043991	<0.001	<1.00E-05
GO:0019882	Antigen processing and presentation	0.608085	2.039404	<0.001	<1.00E-05
GO:0070671	Response to interleukin-12	0.692456	1.97952	<0.001	3.63E-04
GO:0034113	Heterotypic cell-cell adhesion	0.668437	1.968822	<0.001	5.44E-04
GO:1902850	Microtubule cytoskeleton organization involved in mitosis	0.609937	1.926388	<0.001	5.80E-04
GO:0006310	DNA recombination	0.569359	1.97212	<0.001	6.22E-04
GO:0034341	Response to interferon-gamma	0.56863	1.933264	<0.001	6.22E-04
GO:0048285	Organelle fission	0.547311	1.95129	<0.001	6.53E-04
GO:0044839	Cell cycle G2/M phase transition	0.576528	1.939447	<0.001	6.70E-04
GO:0006260	DNA replication	0.56491	1.954217	<0.001	7.25E-04
GO:0032609	Interferon-gamma production	0.613004	1.941457	<0.001	7.25E-04
GO:0032963	Collagen metabolic process	0.637584	1.943146	<0.001	7.91E-04
GO:0051302	Regulation of cell division	0.556745	1.852676	<0.001	8.32E-04
GO:0051383	Kinetochore organization	0.81593	1.85362	<0.001	8.57E-04
GO:0000910	Cytokinesis	0.561381	1.854957	<0.001	8.84E-04
GO:0042113	B cell activation	0.544164	1.856334	<0.001	9.13E-04
GO:0050900	Leukocyte migration	0.528074	1.8591	<0.001	9.43E-04
GO:0071103	DNA conformation change	0.549428	1.847247	<0.001	9.67E-04
GO:0006302	Double-strand break repair	0.552046	1.868762	<0.001	9.76E-04
GO:0007159	Leukocyte cell-cell adhesion	0.523703	1.849113	<0.001	9.95E-04
GO:1902579	Multi-organism localization	0.722924	1.869495	<0.001	0.00101
GO:0032606	Type I interferon production	0.579369	1.873643	<0.001	0.001048
GO:0001539	Cilium or flagellum-dependent cell motility	−0.68717	−1.8666	<0.001	0.043929
GO:0071875	Adrenergic receptor signaling pathway	−0.56796	−1.63536	<0.001	0.192188
GO:0061512	Protein localization to cilium	−0.51386	−1.60418	0.011834	0.194247
GO:0007588	Excretion	−0.45876	−1.53093	<0.001	0.207289
GO:0042219	Cellular modified amino acid catabolic process	−0.5469	−1.47626	0.04142	0.22184
GO:0034394	Protein localization to cell surface	−0.44094	−1.45644	0.017045	0.23749
GO:0051647	Nucleus localization	−0.52992	−1.4452	0.058201	0.238634
GO:0030104	Water homeostasis	−0.43329	−1.48005	0.011765	0.23932
GO:0001578	Microtubule bundle formation	−0.45943	−1.53193	0.006993	0.240921
GO:1901568	Fatty acid derivative metabolic process	−0.38514	−1.48444	<0.001	0.260998
GO:0097503	Sialylation	−0.59227	−1.54516	0.039548	0.263847
GO:0006631	Fatty acid metabolic process	−0.38711	−1.63982	<0.001	0.269063
GO:0031163	Metallo-sulfur cluster assembly	−0.54453	−1.39581	0.102564	0.316266
GO:0044282	Small molecule catabolic process	−0.32625	−1.36148	<0.001	0.327921
GO:0006520	Cellular amino acid metabolic process	−0.33541	−1.36993	<0.001	0.328733
GO:0042737	Drug catabolic process	−0.36808	−1.37799	<0.001	0.333584
GO:0015695	Organic cation transport	−0.45763	−1.3413	0.080214	0.335795
GO:1901615	Organic hydroxy compound metabolic process	−0.28651	−1.2461	<0.001	0.342991
GO:0071867	Response to monoamine	−0.42204	−1.25682	0.176471	0.349968
GO:0007031	Peroxisome organization	−0.3808	−1.32778	0.072464	0.350996
GO:0006790	Sulfur compound metabolic process	−0.29579	−1.24715	0.021277	0.351097
GO:0033865	Nucleoside bisphosphate metabolic process	−0.35745	−1.34247	<0.001	0.353529
GO:0016042	Lipid catabolic process	−0.30883	−1.25087	0.04	0.353532
GO:0042398	Cellular modified amino acid biosynthetic process	−0.40655	−1.25825	0.095541	0.358568
GO:0030258	Lipid modification	−0.32067	−1.31622	<0.001	0.360009

*ES, enriched score; NES, net enriched score; FDR, false discovery rate.*

## Discussion

Comprehensive therapies for non-small-cell lung cancer have improved with the development of precision medicine (Politi and Herbst, 2015). However, lung cancer is still the most fatal disease caused by malignancies. Advances in interpreting NSCLC genomics have boosted progress in understanding specific molecular subsets and refining individual precision treatment (Zappa and Mousa, 2016). Tremendous efforts have been made to increase the lung cancer patient survival rate via the discovery of novel therapeutic targets and prognostic markers (Chang et al., 2015). The latest version of molecular therapy recommends EGFR or BRAF mutations, ALK or ROS1 rearrangements and immunohistochemistry evaluation of PD-L1 without those marker alterations (Lindeman et al., 2018; Camidge et al., 2019; Zhang et al., 2019). These achievements have significantly increased the survival time of lung cancer patients with aberrant genes (Raparia et al., 2013). However, as only a small number of patients qualify for molecular therapy in clinical practice, there is urgent demand to discover novel targets for NSCLC (Vallieres et al., 2012).

PEPT1 (SLC15A1) has been widely studied in cancers and anticancer drug transportation. Several reports have indicated that it could be used as a therapeutic target to enhance doxorubicin efficacy for hepatocellular carcinoma (Gong et al., 2017). In addition, the increasing PEPT1 expression level facilitates 5-fluorouracil treatment in gastric cancer (Inoue et al., 2005). Moreover, PEPT1 was found to be associated with colitis-associated tumorigenesis in a PEPT1-KO mouse model. The overexpression of PEPT1 could reverse the tumorigenesis process, indicating that PEPT1 is a potential therapeutic target for colorectal cancer (Viennois et al., 2016). Interestingly, the PEPT1 expression level was proven to be a major factor for porphyrin accumulation in ALA-PDD and ALA-photodynamic therapy in bladder tumors (Hagiya et al., 2013). Regarding prostate cancer, PEPT1, as well as PEPT2 (SLC15A2), were found to be expressed in the prostate cancer cell lines PC-3 and LNCaP, respectively, and further controlled the cellular uptake of Gly-Sar and L-histidine, indicating their role in the tumor metabolism process (Tai et al., 2013). Due to these transmembrane transport characteristics, Mitsuoka et al. synthesized two novel dipeptides [l-phenylalanyl sarcosine (Phe-Sar) and 4-(4-methoxyphenyl)-l-phenylalanyl sarcosine (Bip(OMe)-Sar)] and successfully inhibited the human pancreatic cancer cell line AsPC-1, demonstrating that PEPT1 has a promising role as an anti-pancreatic cancer progression target (Mitsuoka et al., 2010). A similar result was also observed in the glioma cell line U373-MG for transportation of dipeptide by PEPT2 (Zimmermann et al., 2010). For hepatocellular carcinoma (HCC), whole-genome sequencing analysis revealed that single-nucleotide variations (rs2257212) of SLC15A2 demonstrated better progression-free survival and regulated the sorafenib treatment response (Lee et al., 2015). PEPT1/2 were also found to regulate melatonin intracellular trafficking in PC3 and U118 cells, suggesting their important role in the cancer metabolism delivery system (Huo et al., 2017). SLC15A3 has not been widely studied. However, its impacts on immune macrophage cells were discovered by activation of NF-kB, MAPK, and IRF3 through Toll-like receptors (Song et al., 2018). SLC15A4 also participates in the pathogenesis of lupus, which is known as an immune system disorder (Baccala et al., 2013). More investigations have focused on SLC15A4 regulating TLR-triggered IRF7-IFN-I axis in autoimmune diseases (Kobayashi et al., 2014). SLC15A4 is required for Toll-like receptor 7 (TLR7)- and TLR9-mediated type I interferon (IFN-I) production in plasmacytoid dendritic cells (pDCs) by the mTOR pathway, highlighting its role in regulating the immune response in the tumor microenvironment (Kobayashi et al., 2014). Unlike SLC15A1 and SLC15A2, which are frequently studied in the cancer field, the roles of SLC15A3 and SLC15A4 in cancer are quite opaque. For SLC15A4, only a few studies have shown a very limited understanding of its functions in cancer. For example, the mRNA level of SLC15A4 is increased in the majority of colorectal cancers, and the detection of SLC15A4 and CD44 in feces may help to identify initial CRC cells (Lee et al., 2016). hPHT1 protein is expressed in different intestinal regions. Histidine and carnosine uptake were linear in hPHT1-COS-7 cells over 15 min and were found to be pH-dependent (Bhardwaj et al., 2006). In prostate cancer, SLC15A4 was highly expressed in certain prostate cancer cell lines. However, the exact role of SLC15A4 was not understood compared with SLC15A1 and SLC15A2 (Tai et al., 2013). Notably, SLC15A4 is hypermethylated in A2780CP cells, indicating its role in chemotherapy for ovarian cancer (Yu et al., 2011). An important recent finding showed that siRNA-mediated knockdown of PHT1 could significantly reduce the uptake of carnosine in glioblastoma cells, providing evidence *in vitro* that PHT1 could inhibit the growth of tumor cells by affecting carnosine uptake (Oppermann et al., 2019). However, there are no reports on these gene network functions in lung cancer or tumor prognosis prediction biomarkers. It is crucial to intensively explore the role of SLC15A4 in malignancies, as the understanding of SLC15A4 in cancer is quite limited, and its physiological and pharmacological status is unknown.

Our results showed that SLC15A2 and SLC15A3 RNA expression levels were decreased in lung cancer tissues compared with normal tissues by Oncomine pan-cancer analysis. The RNAseq expression of SLC15A2, SLC15A3, and SLC15A4 were consistent with Oncomine result. However, compared with normal lung tissue, none of the three genes were decreased with a significant statistical difference. This indicates that SLC15A family may not be a good marker to distinguish the origin of cancer or used to be an early detection maker before the cancer phenotypes. Interestingly, all four gene members of the SLC15A family could determine NSCLC overall prognosis independently, but none of them could be used as a prognostic marker for SCC. Only SLC15A2 and SLC15A4 were suitable to predict the clinical outcome of lung adenocarcinoma. Notably, there is an inverted trend of SLC15A1 in overall survival prediction after 140 months follow-up, which may be majorly due to the greatly decrease of the patient number. We should pay attention that if the patient number is small, the result may be even the opposite. As SLC15A2 RNA-seq expression is ultra-low and intensively studied in cancer, we aimed to reveal more molecular functions of SLC15A4. We identified SLC15A4-correlated genes from TCGA-LUAD and further investigated these gene enrichment functions. Our results show that SLC15A4 mainly participated in the cell cycle and division, implying its role in cancer proliferation. Additionally, the metabolic pathways including fatty-acid and lactate were enriched as expected according to the literature. As a PH-sensitive transporter, the lactate metabolic cycle may greatly get involved. These pathway enrichments highlight that SLC15A4 may have major functions through peptide intake to control the cell cycle, which directly exerts a phenotype in cancer cell proliferation. Taking these results into consideration, the major cell cycle effector proteins, such as cyclin A, B, D, and F or the CDK family, should be tested to verify SLC15A4 function. Moreover, the MAPK/ERK pathway, which could be affected by arachidonic acid through SLC15A4, is also worthy of investigation. As other researchers have found that SLC15A4 may have a great impact on the mTOR pathway, the functions of SLC15A4 in the proliferation of lung cancer cells should also be revealed. GSEA showed that SLC15A4 mainly positively participated in chromosome segregation and negatively regulated cilium- or flagellum-dependent cell motility. The IHC staining pattern showed that the SLC15A4 protein level could be distinguished by a pathologist, as the patients may have strong membrane staining and strong cytoplasmic intensity in the cancer cells. As the normal lung tissue contains macrophages with a similar staining pattern, the clinical practice should be well noted. Taken together, our findings clearly demonstrated that SLC15A4 could be an ideal lung adenocarcinoma prognostic marker and could be further targeted.

The main challenge for lung cancer is to identify the population of patients who can benefit from targeted treatment; thus, there is an urgent need for effective indicators (Custodio et al., 2012). The purpose of our study was to understand the clinical value and molecular mechanism of the SLC15A family in lung cancer through comprehensive bioinformatics analysis. At the same time, we hope that our findings for SLC15A4 will provide new prospects for future research and clinical application in lung cancer patients. The transportation of various substrates including small molecular drugs highlights the relational design of pharmacological compounds for delivery through SLC15A4.

It draws attention that the dipeptide-mimetic bestatin, which is known as an anti-cancer substrate transported by SLC15A4, may enhance its function via up-regulating SLC15A4 activity (Harada et al., 1998). More importantly, the regulation of SLC15A4 transportation activities is also promising for anti-cancer research. As the expression level of SLC15A4 is highly associated with its activity, the inhibitor or activator drugs of SLC15A4 are required to explore their functions in lung cancer, as well as the gene-editing tool. Nateglinide and glibenclamide could block the peptide transporters with high affinity (Terada et al., 2000). Other drugs designed on basis of lysyl-dipeptides as high-affinity-type competitive inhibitors for other members in SLC15A family may affect the function of SLC15A4 (Theis et al., 2002). However, their exact role in cancer was not tested. According to our study, more investigations should be performed to validate SLC15A4 as a novel indicator or drug target for lung adenocarcinoma in translational medicine.

## Data Availability Statement

The original contributions presented in the study are included in the article/Supplementary Material, further inquiries can be directed to the corresponding author/s.

## Ethics Statement

The studies involving human participants were reviewed and approved by the study was approved by the Ethics Committee of Harbin Medical University. The patients/participants provided their written informed consent to participate in this study.

## Author Contributions

HH, JW, and SC drafted the manuscript and analyzed the data. HH, YS, XG, LG, and JJ performed figure preparation and data analysis. MG performed critical revision of the whole work. SY and HC performed research design, manuscript drafting, and revision. All authors contributed to the article and approved the submitted version.

## Conflict of Interest

The authors declare that the research was conducted in the absence of any commercial or financial relationships that could be construed as a potential conflict of interest.

## Footnotes

1 www.oncomine.org

2 http://www.cbioportal.org/

3 http://kmplot.com/analysis/

4 https://www.proteinatlas.org/

5 http://www.linkedomics.org/login.php

6 http://metascape.org

## Supplementary Material

Supplementary Figure 1 SLC15A family associations with clinical stages and mutation rate. **(A)** Violin plots of the SLC15A gene family in NSCLC for major stages (TNM stage system). **(B)** The mutation rate in the TCGA-NSCLC data cohort from cBioPortal using TCGA NSCLC 2016 cohort. A1 = SLC15A1, A2 = SLC15A2, A3 = SLC15A3, and A4 = SLC15A4.

Supplementary Figure 2 Volcano plot of all correlated genes of SLC15A4 in TCGA-LUAD with Pearson’s test. Green indicates negatively correlated genes; red indicates positively correlated genes.
