# Supplementary figures and images for: SLC15A4 Serves as a Novel Prognostic Biomarker and Target for Lung Adenocarcinoma
Source: Front Genet. 2021 Jun 8;12:666607. doi: 10.3389/fgene.2021.666607 (PMC8217884; doi:10.3389/fgene.2021.666607)

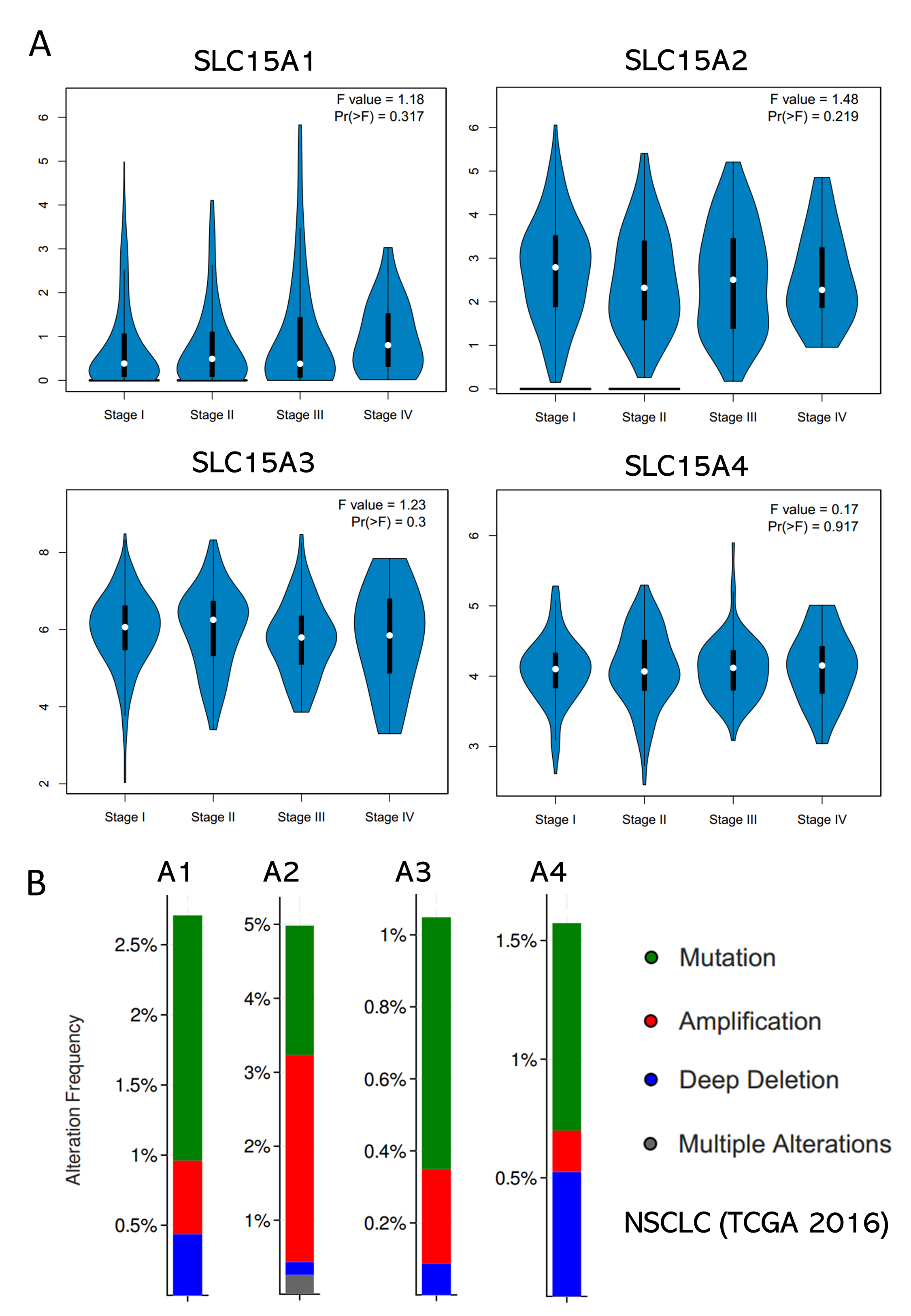

Supplement: Supplementary Figure 1 — SLC15A family associations with clinical stages and mutation rate. (A) Violin plots of the SLC15A gene family in NSCLC for major stages (TNM stage system). (B) The mutation rate in the TCGA-NSCLC data cohort from cBioPortal using TCGA NSCLC 2016 cohort. A1 = SLC15A1, A2 = SLC15A2, A3 = SLC15A3, and A4 = SLC15A4. [file Image_1.TIF]

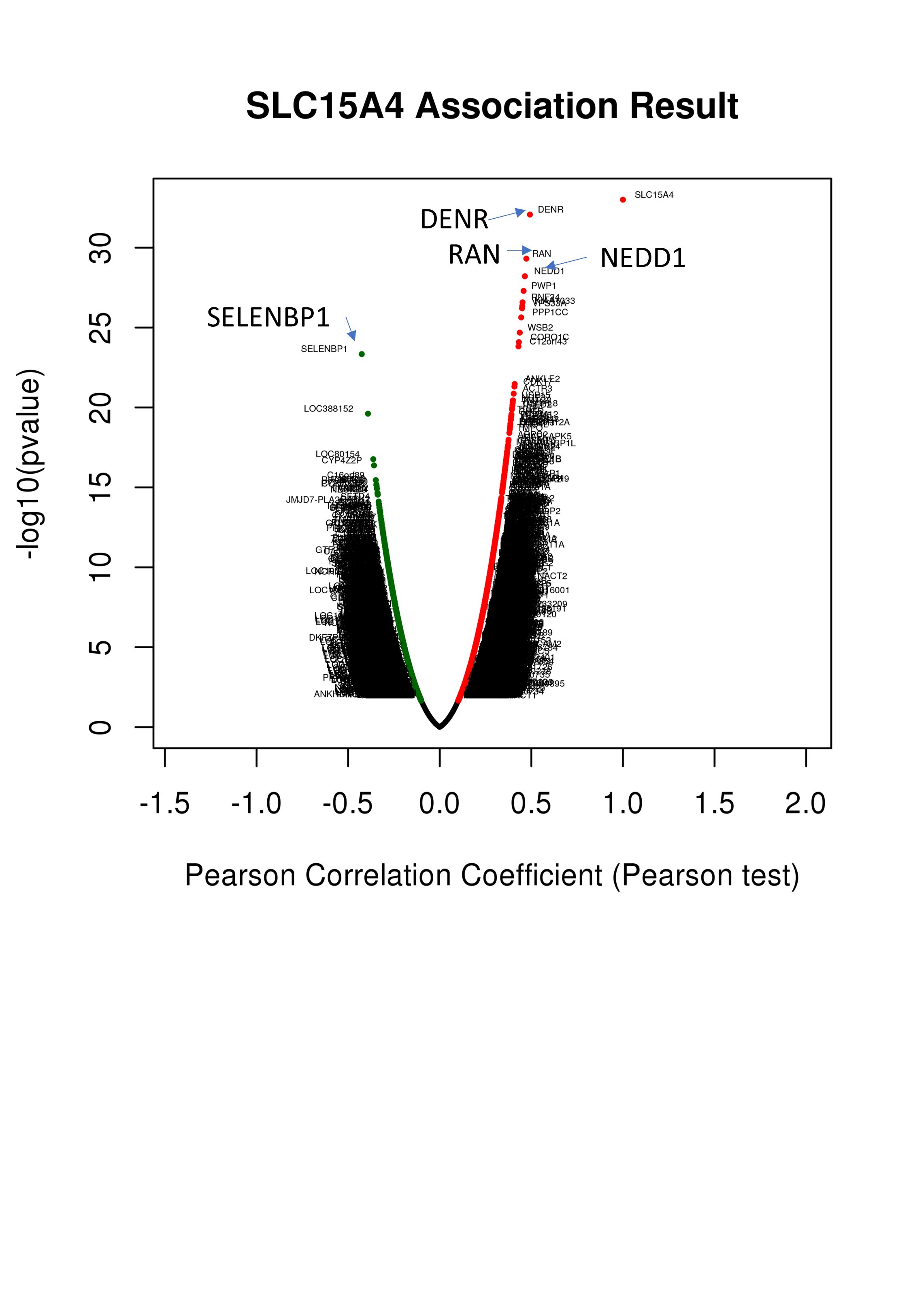

Supplement: Supplementary Figure 2 — Volcano plot of all correlated genes of SLC15A4 in TCGA-LUAD with Pearson’s test. Green indicates negatively correlated genes; red indicates positively correlated genes. [file Image_2.TIF]
